# Socioecological Models of Acculturation: The Relative Roles of Social and Contextual Factors on Acculturation Across Life Domains

**DOI:** 10.3390/bs15060715

**Published:** 2025-05-22

**Authors:** Sara L. Buckingham

**Affiliations:** Department of Psychology, University of Alaska Anchorage, Anchorage, AK 99508, USA; sbuckingham@alaska.edu; Tel.: +1-(907)-786-1767

**Keywords:** acculturation, context of reception, sense of community, intergroup anxiety, symbolic threat, prejudice, community contact, migration, Latine immigrants

## Abstract

Although acculturation research recognizes that a community’s context of reception shapes acculturation, relatively limited research has explored how social and contextual variables shape the divergence of ‘real’ acculturation from ‘ideal’ (or individually desired) acculturation across life domains. Building on the Relative Acculturation Extended Model, this study examines how one’s local context and interactions within it shape acculturation in peripheral (public), intermediate (social), and central (private) life domains. In this cross-sectional study, 408 first-generation immigrant adults born in Latin America (*M* = 37.91, *SD* = 12.93) who had lived in the United States for 1–55 years (*M* = 16.56; *SD* = 9.50) completed self-report measures across four communities with distinct contexts of reception. Results revealed that perception of the receiving community’s acculturation preferences, prejudice, community contact, and symbolic threat all shaped immigrants’ ‘real’ acculturation beyond ‘ideal’, both directly and indirectly via their sense of community and intergroup anxiety. These socioecological factors had a stronger impact on peripheral domain acculturation overall, though pathways generally remained consistent across life domains. Results have numerous implications for policy, practice, and the future of acculturation research as they suggest that social context more readily shapes public expressions of acculturation than more private expressions.

## 1. Introduction

Acculturation—the process by which individuals navigate cultural change and maintenance in the context of intercultural contact—is a complex process shaped by the dynamic interplay between individuals and the sociocultural environments in which they live. Traditionally conceptualized as a psychological phenomenon driven by personal choices and motivations, acculturation is increasingly understood as a process influenced by broader structural and relational factors (Berry & Sam, 2006; Fedi et al., 2019; Schwartz et al., 2010). Indeed, a burgeoning body of research shows that acculturation varies according to one’s experiences in one’s context (Bornstein, 2017). Immigrants’ abilities to enact their preferred cultural practices may be constrained and facilitated by elements in their communities. Understanding how setting factors impact acculturation is essential.

Still, although the field recognizes that contexts of reception (Schwartz et al., 2010) influence acculturation, comparably limited research has examined how social and contextual variables shape the divergence of acculturation behaviors from acculturation attitudes compared to personal factors. To more fully comprehend the phenomenon, we must understand how such malleable variables may affect acculturation. This paper proposes a socioecological model of acculturation in which a set of social and contextual factors—that is, receiving communities’ acculturation preferences and prejudice; concern that receiving communities present a threat to one’s own culture or ‘symbolic threat’; and quality of contact with immigrant and receiving community members predict immigrants’ acculturation outcomes above and beyond their desires, directly and indirectly through their sense of community and intergroup anxiety.

### 1.1. Models of Acculturation

Acculturation is the process by which individuals evaluate and potentially adapt their behaviors, values, beliefs, and cultural identities in response to interaction with different cultural groups (Schwartz et al., 2010). As a concept, contextual influences on acculturation date back to 2370 B.C., when the rulers of Mesopotamia established laws to protect traditional cultural practices from acculturative changes (Rudmin, 2003). The first widely agreed-upon definition of acculturation appeared in the scientific literature in 1936, as “those phenomena which result when groups of individuals having different cultures come into continuous first-hand contact, with subsequent changes in the original culture patterns of either or both groups” (Redfield et al., 1936, p. 149). Acculturation was first predominantly studied as a unidimensional process, where adopting aspects of a new culture was seen as coming at the expense of losing elements of the original one. Bidimensional models later emerged, highlighting that cultural acquisition and cultural retention can coexist. Berry’s (1980) foundational model captures this duality, balancing the desire for contact with members of the ‘majority’ culture against the wish to maintain one’s ‘heritage’ culture, resulting in different acculturation strategies (Berry, 1980). Others, such as Bourhis et al. (1997), have argued that the desire for contact might be better understood as the desire to adopt the majority culture, redefining the core strategies.

The assumption that individuals change and maintain cultures because of personal desires to do so is embedded in much acculturation research (Chirkov, 2009). A host of other individual-level variables have also been linked to acculturation, among them age, socioeconomic status, personality, social skills, and resilience (Berry & Sam, 2006). As the literature expands, it has become clear that socioecological contexts and processes also impact acculturation (Bornstein, 2017; Horenczyk et al., 2015). Indeed, more recent conceptualizations of acculturation have emphasized the difference between acculturation behaviors, or what one does, and attitudes, or what one thinks or prefers (e.g., Arends-Tóth et al., 2006; Birman et al., 2005).

One such contemporary model, the Relative Acculturation Extended Model (RAEM; Navas et al., 2005), differentiates between ‘ideal’ and ‘real’ acculturation. *Ideal acculturation* reflects an attitude or the ways in which people desire to change and maintain cultures. *Real acculturation* reflects strategies put into practice, or the ways in which people actually do change and maintain their cultures. For example, a parent may hope to uphold their heritage child-rearing practices, such as collective caregiving or specific discipline procedures, but find themselves adjusting to the norms of the new society—perhaps due to school expectations, legal frameworks, or their children’s own preferences in this new place. On the other hand, that parent might wish to develop friendships with members of the receiving community but instead socialize primarily with other immigrants from their country of origin—perhaps because of language barriers, exclusion, or practical constraints. The RAEM proposes that individuals may enact cultures differently according to the life domain. The *peripheral* domain includes such public-facing cultural elements of employment, economics/consumption, political/government systems, and social welfare systems. The *intermediate* domain involves social relationships and friendships. The *central* domain comprises more private aspects of life, including family relationships, religious customs, ways of thinking and principles/values (Navas et al., 2005). The RAEM acknowledges that people may have different expectations for how others should acculturate; that is, they prefer for others to change and maintain their cultures in certain ways, and they may or may not perceive people to be acculturating in these ways (Navas et al., 2007). Cultural change and maintenance can be analyzed together, allowing for the identification of categorical acculturation strategies (e.g., assimilation, separation, marginalization, integration/biculturalism) or examined as dimensions on a continuum.

### 1.2. A Socioecological Model of Acculturation

Available or expected options within one’s local context and interactions within it may shape ‘real’ acculturation. This is encapsulated in the concept of *context of reception*, or the aspects of a community that shape a newcomer’s experience, such as policies within a community that affect daily life, social reception by longer-term residents, and access to and treatment by community institutions (Portes & Rumbaut, 2024; Schwartz et al., 2014). When immigrants resettle, they encounter multiple nested contexts of reception—neighborhood, municipality, state, nation—that together shape their experiences (Perez, 2021). Due to their immediate impact, smaller, local contexts can often have as much or even greater influence on immigrants’ experiences than broader national contexts (Buckingham et al., 2018a, 2018b; Ellis & Almgren, 2009; Golash-Boza & Valdez, 2018). Most contexts of reception are not purely positive or negative; rather, they present both support and challenges (Schwartz et al., 2014).

With the context of reception as an organizing framework, the literature reveals several community-level factors that are likely to shape acculturation. The *receiving community’s preferences* for immigrants’ cultural change and maintenance (Navas et al., 2005, 2007) along with better *quality of contact* (Allport, 1954; Amir, 1969) with both immigrants and receiving community members likely to promote acculturation (Buckingham et al., 2018a, 2018b; Fedi et al., 2019). On the other hand, experiencing *prejudice and discrimination* (e.g., xenophobia; Yakushko, 2009) from the receiving community may impede cultural change while promoting cultural maintenance by prompting immigrants to turn away from national-born citizens and turn more solely to immigrant communities to fulfill needs (e.g., Dandy & Pe-Pua, 2013; Valdez et al., 2013). Finally, *symbolic threat*, or belief that different morals/values/beliefs/attitudes/standards put one’s own beliefs/values/worldviews at risk (Stephan & Stephan, 2000) may affect how immigrants perceive and respond to receiving community members—as it does for receiving community members (e.g., Moftizadeh et al., 2021; Vezzali & Giovannini, 2010)—thus impeding change and promoting maintenance.

While each of these factors may impact acculturation directly, the literature suggests two psychosocial processes may explain how each shapes acculturation: Psychological sense of community (PSOC) and intergroup anxiety. PSOC refers to one’s *membership*, belonging, and identification with a community; *shared emotional connection* from community history and bonds; *integration and fulfillment of needs* through intangible and tangible resources received from membership; and *mutual influence* of the community and its members on one another (McMillan & Chavis, 1986). Through migration, people can develop PSOC to multiple communities, such as a territorial receiving community and an ethnic immigrant community (Bathum & Baumann, 2007; Mannarini et al., 2018; Maya-Jariego, 2006). PSOC is promoted through experiences like positive contact and attitudes of community members (Barmania, 2015; Malsbary, 2014; Maya-Jariego & Armitage, 2007), and its development is likely to promote the norms of the community with which it is experienced, related to social cohesion, social identity, meaning and adaptation (Mannarini et al., 2018; Obst & White, 2005; Sonn, 2002; Townley et al., 2011). Consistent with this, frameworks such as *rejection-identification* and *rejection-disidentification* suggest that perceived exclusion may reinforce affiliation with one’s heritage community or diminish connection with the receiving community, components of PSOC (Branscombe et al., 1999; Jasinskaja-Lahti et al., 2009). Further, *intergroup anxiety*—negative cognitions, emotions, and behaviors that emerge from actual or anticipated contact with community members who diverge on some characteristic, such as migration status—developed through attitudes and experiences including symbolic threat, prejudice, and negative contact, likely impedes cultural change (Binder et al., 2009; Stephan, 2014; Turner et al., 2008).

### 1.3. Current Study

In this study, I examine whether socioecological models of acculturation better explain immigrants’ acculturation outcomes (‘real’ acculturation) above and beyond their desires (‘ideal’ acculturation). First, I hypothesize that perceived preferences for cultural change and quality of contact with the receiving community will be positively associated with real cultural change, both directly and indirectly through increased PSOC with the receiving community and decreased intergroup anxiety, whereas prejudice and symbolic threat will be negatively associated with real cultural change, both directly and indirectly through decreased PSOC with the receiving community and increased intergroup anxiety. Second, I hypothesize that perceived preferences for cultural maintenance, prejudice, quality of contact with the ethnic immigrant community, and symbolic threat will all be positively associated with real cultural maintenance, both directly and indirectly through increased PSOC with the ethnic immigrant community. Finally, I hypothesize that socioecological variables will explain progressively more variance in central, intermediate, and peripheral domains, as acculturation in more public life domains likely requires more cooperation from others to be enacted.

## 2. Materials and Methods

### 2.1. Context

Data comes from two U.S. regions: Mid-Atlantic (Maryland and Virginia) and Southwest (New Mexico and Arizona). Participants were recruited from urban areas: Baltimore, Richmond, Albuquerque, and Phoenix. Each is distinct, with communities within each region sharing similar population demographics (such as their foreign-born population make-up and immigration patterns) while having divergent immigration-related public policies, likely creating very different contexts of reception for Latine immigrants.

### 2.2. Participants and Participant Recruitment

To be eligible, participants had to have emigrated from a Spanish-speaking Latin American country, be at least age 18, speak English or Spanish, and live in Maryland, Virginia, New Mexico, or Arizona. We used a stratified snowball sampling approach to recruit this somewhat hard-to-reach population during the first Trump bid for presidency, in 2015 (Martinez et al., 2012). Local researchers and community partners recruited participants at locations serving Latine immigrants and at broader community spaces like markets, places of worship, sports leagues, and festivals. We shared information in both print and audio formats, providing full information about the study, communicating in participants’ preferred languages, answering questions, and providing contact information. In all, 65.0% of participants encountered the project through researchers, 13.5% through a community organization, 13.7% through friends or family, and 7.7% through other sources (e.g., online, flyers, radio, articles).

The final sample was 408 (Arizona = 99, Maryland = 114, New Mexico = 99, Virginia = 96). A little over half were born in Mexico, while a quarter came from Central America and a fifth from South America; only a small number were from the Caribbean. Participants ranged in age from 18 to 77, with immigration occurring across life stages. Length of U.S. residence varied from less than one year to 55 years. Common reasons for immigration included seeking a better quality of life, economic opportunities, employment, education, safety, and family reunification. Women were overrepresented. Nearly 4/10 had obtained U.S. citizenship, 3/10 had a legal immigration status, and the remaining 3/10 lacked authorization or did not disclose their status (see Table 1).

### 2.3. Procedures and Instruments

Following an introduction to the study, informed consent, eligibility screening, and answers to questions, participants completed an online or paper survey in English or Spanish. Participants with low literacy (*n* = 7) responded to items aloud and researchers recorded their responses. They could enter a raffle for one of thirteen $30–$100 Visa gift cards and indicate interest in a focus group to discuss topics further. Participants were debriefed and provided follow-up information at the end conclusion of the interaction.

#### 2.3.1. Acculturation

Acculturation was measured with the 32-item RAEM Scale (Navas et al., 2005), responded to on a 5-point Likert scale, ranging from 1 (“not at all”) to 5 (“a lot”). For real cultural change, participants were asked, *“To what extent have you adopted U.S./‘American’ culture?”* across the domains: *peripheral* (work, economics, politics, social welfare), *intermediate* (social relations), and *central* (family relations, religious customs, beliefs/values). For real cultural maintenance, they were asked, *“To what extent have you maintained your original culture?”* across domains. For ideal cultural change and maintenance, they were asked, *“When you first arrived in the United States*, *to what extent did you want to adopt U.S./‘American’ culture* [change]/*maintain your original culture* [maintenance]*?”* across domains. Internal consistency was acceptable to good for each subscale (α = 0.74–0.81). Responses for items on each domain subscale were averaged, ranging from 1 to 5, with higher scores indicating greater acculturation; domain scores were then summed to create total scores (see Table 2).

#### 2.3.2. Psychological Sense of Community (PSOC)

PSOC was measured with the 24-item Sense of Community Index, Second Edition (Chavis et al., 2008), which uses a 4-point Likert scale, ranging from 1 (“not at all”) to 4 (“completely”). Participants responded once in reference to their broader local receiving community (α = 0.97), and once in reference to their ethnic (Latine) immigrant community (α = 0.95). Responses were summed for each community, ranging from 24 to 96, with higher scores indicating stronger PSOC (see Table 2).

#### 2.3.3. Intergroup Anxiety

Intergroup anxiety was measured with the 12-item Intergroup Anxiety Scale–Modified (Stephan et al., 2002a), which uses a 10-point Likert scale, ranging from 1 (“not at all”) to 10 (“extremely”). It consists of items regarding how one feels (e.g., uncertain, safe) interacting with members of the receiving community (α = 0.90). Positive emotions were reverse-coded before summing. Scored ranged from 12 to 106, with higher scores indicating more intergroup anxiety (see Table 2).

#### 2.3.4. Perceived Acculturation Preferences

Perceived acculturation preferences of the receiving community for immigrants were measured with the 16-item ideal subscale of the receiving community version of the RAEM measure (Navas et al., 2005), which was responded to on a 5-point Likert scale, ranging from 1 (“not at all”) to 5 (“a lot”). Participants were asked, *“To what extent does the United States want you to … adopt U.S./‘American culture’* [change] and *“… maintain your original culture”* [maintenance] across the domains specified above. Responses for items on each domain subscale were averaged, ranging from 1 to 5, with higher scores indicating greater acculturation; domains were then summed to create total scores (α = 0.85 for each subscale, see Table 2).

#### 2.3.5. Prejudice

Prejudice was measured with the 18-item public subscale of the Perceived Racism Scale for Latina/os (Collado-Proctor, 1999; Moradi & Risco, 2006), which was slightly modified to reflect immigrant status, replacing the original subject (“Latino”) with “Latina/o immigrant.” The public subscale includes both overt and covert prejudicial attitudes and discrimination experienced over the past year, responded to on a 5-point Likert scale, ranging from 0 (“never”) to 4 (“several times a day”) along with a not applicable (n/a) option for experiences participants had not had in the past year and thus could not have experienced prejudice in that experience (e.g., those who never had applied for a loan could select “n/a” to the question “Because I am a Latina/o immigrant, I have been turned down for loans”) (α = 0.94). After removing n/a responses, the remaining items were summed to yield an overall frequency of perceived prejudice and discrimination score, with higher scores indicating greater frequency. Scores ranged from 0 to 66 (see Table 2).

#### 2.3.6. Quality of Contact

Quality of contact with U.S.-born people and Latine immigrants was modeled after Castellini et al.’s (2011) approach. Participants were asked to assess the quality of their contact with Latine immigrants and U.S.-born Americans as friends, neighbors, school/work colleagues, and people in public places by responding to eight items on a 5-point Likert scale, ranging from 1 (“very negative”) to 5 (“very positive”). Responses were summed for each subscale, with higher scores indicating more positive contact. Scores ranged from 4 to 20 (U.S.-born: α = 0.84, Latine immigrant: α = 0.81; see Table 2).

#### 2.3.7. Symbolic Threat

The 12-item Symbolic Threats Measure (Stephan et al., 2002b) was used to assess threats stemming from perceived value and belief differences with receiving community members, responded to on a 7-point Likert scale, ranging from 1 (“strongly disagree”) to 7 (“strongly agree”). Responses were summed with higher scores indicating more perceived threat (α = 0.93). Scores ranged from 12 to 84 (see Table 2).

#### 2.3.8. Demographic Measures

Measured demographic characteristics included zip code, country of origin, gender, age, age at immigration, race, motivation(s) for immigration, household members, other family members in the U.S., family members in country of origin, income, education, employment, and immigration status.

### 2.4. Analyses

Prior to analysis, data were screened for missingness and found to be minimal overall (<10%) and not systematic in the final sample. For scale-based variables, mean scores were calculated when at least 80% of items were completed, consistent with best practices (Graham, 2009). In cases where these scale scores are commonly reportedly as sums in the literature, the means were then multiplied by the number of items on the scale to produce the sum (see Table 2). 

All analyses were performed in IBM SPSS Statistics Version 29; main analyses were conducted via hierarchical regression-based path analysis based on procedures described by Hayes (2013).

## 3. Results

### 3.1. Main Path Models

**Figure 1:**
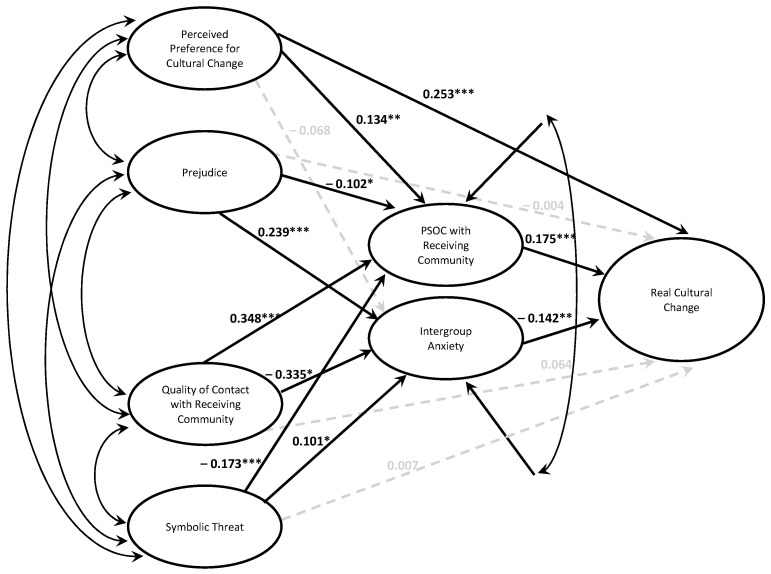
Socioecological model of the overall cultural change. *Note.* Model controls for ideal cultural change, time in the U.S., and immigration status. * *p* < 0.05, ** *p* < 0.01, *** *p* < 0.001.

A hierarchical regression analysis was conducted to evaluate whether socioecological variables explained additional variance in real cultural change beyond an individual-focused model. The first step included ideal cultural change, immigration status^1^, and time in the U.S., which accounted for a significant proportion of variance in real cultural change. The individual-level predictors controlled for 29.8% of the variance in real cultural change [*F*(3, 404) = 57.26, *p* < 0.001]. In the second step, perceived preference for cultural change, prejudice, quality of contact with the receiving community, and symbolic threat were entered into the model, which accounted for 14.5% more variance [Δ*F*(6, 398) = 17.13, *p* < 0.001]. As hypothesized, the more participants perceived the receiving community to desire cultural change, the more cultural change they reported, partially mediated through PSOC. The better the quality of contact participants reported with the receiving community, the more PSOC they experienced and the less intergroup they experienced, which in turn was associated with more cultural change. On the other hand, the more prejudice and symbolic threat participants perceived, the less PSOC they experienced and the more intergroup anxiety they experienced, which in turn was associated with less cultural change; PSOC and intergroup anxiety fully mediated the relation between symbolic threat, prejudice, and cultural change (see Figure 1 for the path model and Table 3 and Table 4 for tests of the path coefficients). The full set of variables accounted for 44.4% of the variance in cultural change [*F*(9, 398) = 35.25, *p* < 0.001].

**Figure 2:**
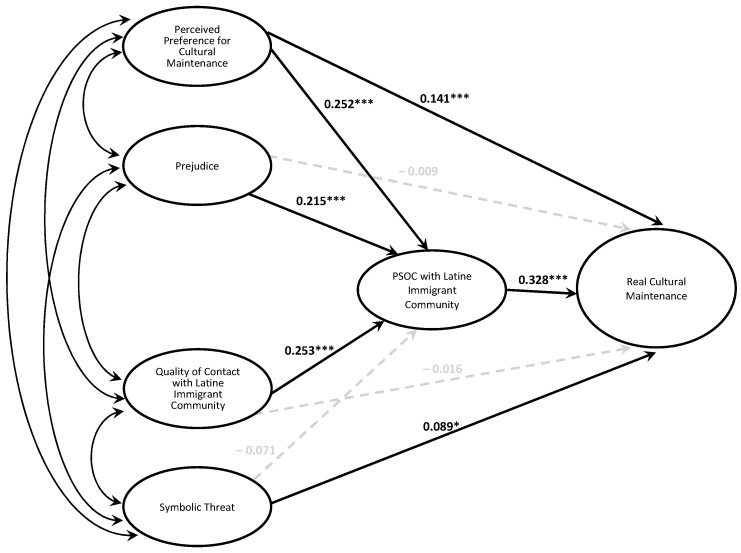
Socioecological model of overall cultural maintenance. *Note.* Model controls for ideal cultural maintenance. * *p* < 0.05, ** *p* < 0.01, *** *p* < 0.001.

A second hierarchical regression analysis was conducted to evaluate whether socioecological variables explained additional variance in real cultural maintenance beyond an individual-focused model. The first step included ideal cultural maintenance^2^, which accounted for 28.1% of the variance in real cultural maintenance [*F*(1, 406) = 158.53, *p* < 0.001]. At the second step, the receiving community’s perceived preference for cultural maintenance, prejudice, quality of contact with Latine immigrants, and symbolic threat were entered into the model, accounting for 13.7% more variance [Δ*F*(5, 401) = 18.83, *p* < 0.001]. Whereas prejudice and quality of contact with Latine immigrants were only indirectly positively associated with cultural maintenance through increased PSOC with the Latine immigrant community, and symbolic threat only was positively directly associated with cultural maintenance, the receiving community’s perceived preference for cultural maintenance both directly and indirectly promoted cultural maintenance through PSOC with the Latine immigrant community (see Figure 2 for the path model and Table 5 and Table 6 for tests of the path coefficients). In all, the predictors accounted for 40.9% of the variance in cultural maintenance [*F*(6, 401) = 47.92, *p* < 0.001].

### 3.2. Domain Models

Socioecological variables were then examined to see whether they accounted for more variance in acculturation in particular life domains (peripheral, intermediate, central).^3^

#### 3.2.1. Peripheral

**Figure 3:**
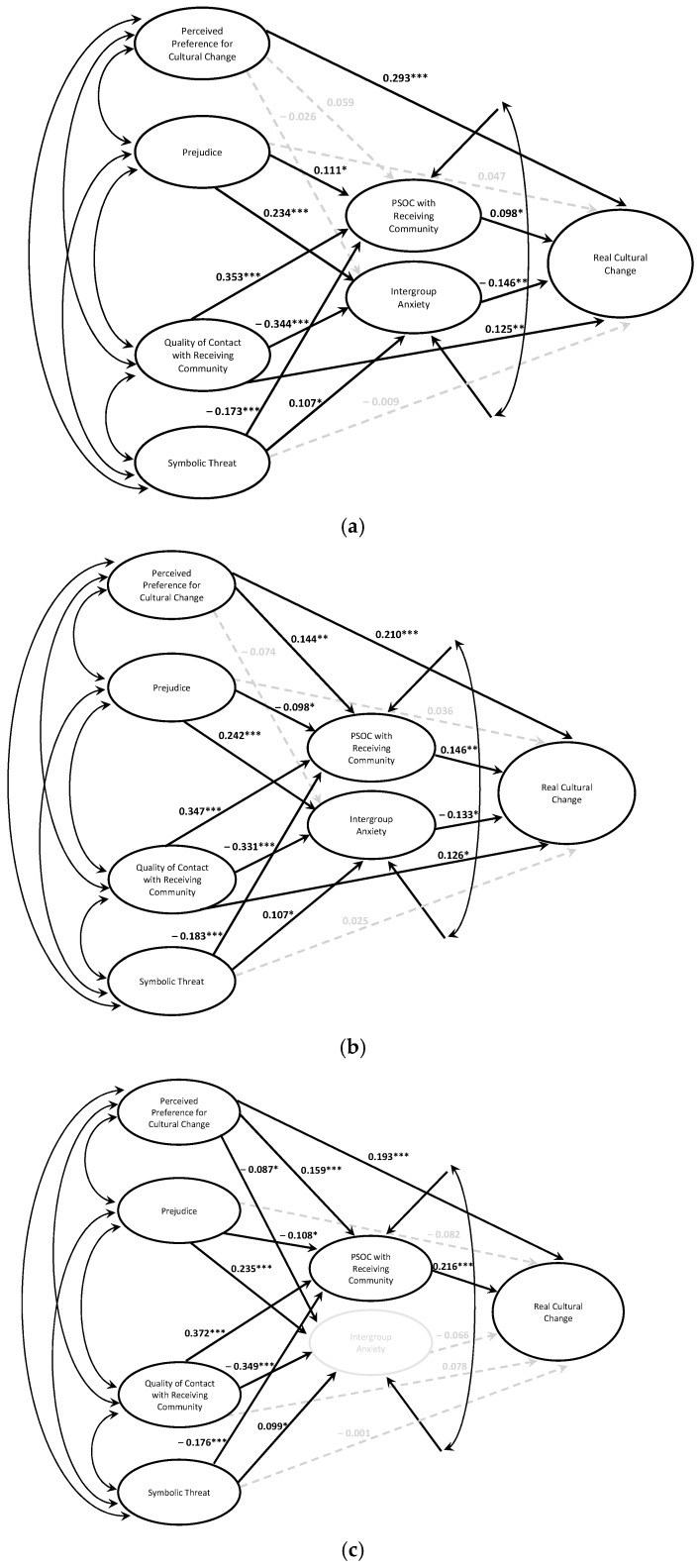
Socioecological models of cultural change across domains. (**a**). Peripheral. (**b**). Intermediate. (**c**). Central. *Note.* Models control for ideal cultural change, time in the U.S., and immigration status. * *p* < 0.05, ** *p* < 0.01, *** *p* < 0.001.

The socioecological variables accounted for an additional 14.9% of variance in peripheral domain cultural change beyond ideal [Δ*F*(6, 398) = 18.64, *p* < 0.001]. The peripheral domain model (see Figure 3a) matched the overall cultural change model with two exceptions: (a) perceived preference for cultural change was only directly associated with peripheral domain cultural change and (b) quality of contact with the receiving community had both direct and indirect associations with peripheral domain cultural change. The full set of variables accounted for 47.0% of the variance in peripheral domain cultural change [*F*(9, 398) = 39.14, *p* < 0.001].

**Figure 4:**
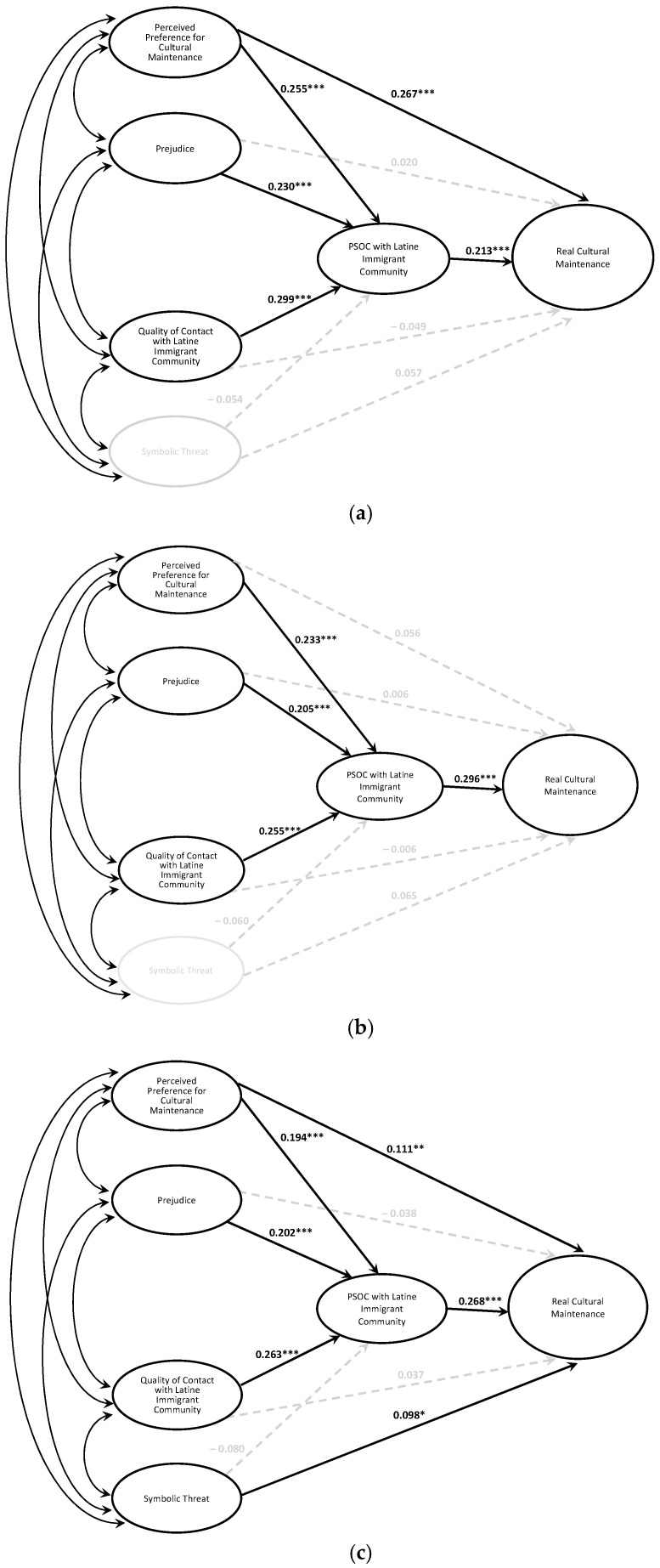
Socioecological models of cultural maintenance across domains. (**a**). Peripheral. (**b**). Intermediate. (**c**). Central. *Note.* Models control for ideal cultural maintenance. * *p* < 0.05, ** *p* < 0.01, *** *p* < 0.001.

The socioecological variables accounted for an additional 12.7% of the variance in peripheral domain cultural maintenance beyond ideal [Δ*F*(5, 401) = 18.14, *p* < 0.001]. While the domain model (see Figure 4a) matched the overall cultural maintenance model, symbolic threat was not significantly associated with peripheral domain cultural maintenance. The full set of variables accounted for 44.0% of the variance in peripheral domain cultural maintenance [*F*(6, 401) = 52.49, *p* < 0.001].

#### 3.2.2. Intermediate

The socioecological variables accounted for an additional 12.7% of variance in intermediate domain cultural change beyond ideal [Δ*F*(6, 398) = 12.99, *p* < 0.001]. The intermediate domain (see Figure 3b) matched the overall model, with one exception: quality of contact with the receiving community has both direct and indirect associations with intermediate domain cultural change. The full set of variables accounted for 35.0% of the variance in intermediate domain cultural change [*F*(9, 398) = 23.80, *p* < 0.001].

The socioecological variables accounted for an additional 9.6% of variance in intermediate domain cultural maintenance beyond ideal [Δ*F*(5, 401) = 10.66, *p* < 0.001]. The intermediate domain model (see Figure 4b) matched the overall cultural maintenance model with two exceptions: (a) symbolic threat was not significantly associated with cultural maintenance, and (b) perceived preference for cultural maintenance was only indirectly associated with peripheral domain cultural maintenance through PSOC with the Latine immigrant community. The full set of variables accounted for 27.6% of the variance in intermediate domain cultural maintenance [*F*(6, 401) = 25.47, *p* < 0.001].

#### 3.2.3. Central

The socioecological variables controlled for an additional 10.0% of variance in cultural change beyond ideal [Δ*F*(6, 398) = 11.32, *p* < 0.001]. In the central domain model (see Figure 3c), only PSOC served as a mediator; that is, intergroup anxiety did not control for a significant amount of variance in central domain cultural change. The full set of variables accounted for 41.2% of the variance in central domain cultural change [*F*(9, 398) = 30.96, *p* < 0.001].

The socioecological variables controlled for an additional 10.1% of variance in cultural maintenance beyond ideal [Δ*F*(5, 401) = 12.53, *p* < 0.001]. The central domain model (see Figure 4c) matched the overall cultural maintenance model, with the full set of variables accounting for 35.4% of the variance in central domain cultural maintenance [*F*(6, 401) = 36.62, *p* < 0.001].

## 4. Discussion

Results indicate that we must continue to look beyond the individual to fully understand acculturation. Individuals, their contexts, and their interactions influence acculturation (Berry & Sam, 2006; Bornstein, 2017; Chirkov, 2009; Horenczyk et al., 2015). While individual desire (‘ideal’ acculturation) accounted for the single most amount of variance in acculturation strategies (‘real’ acculturation), the set of social and contextual variables controlled for between approximately 10 and 15% more variance depending on life domain, allowing for a prediction of up to nearly half of the variance in acculturation.

The strength of the socioecology’s influence varies, to some degree, across life domains. The socioecological variables considered here generally controlled for more variance in peripheral domain acculturation than central domain acculturation. This may be related to central domain acculturation being more within an individual’s control and less dependent on others. For example, immigrants may more easily practice their desired religious customs and enact their preferred values in ways that do not necessarily require others’ support; likewise, there are likely less frequent or imposing barriers to central domain acculturation aspects of worldviews, principles, beliefs, and family relationships. This is further supported by empirical data that shows that, in contrast to the peripheral and intermediate domains, intergroup anxiety did not account for a significant amount of variance in central domain cultural changes. On the other hand, immigrants need others to enact desired cultural practices in social relationships and employment, for example, as acculturation in these intermediate and peripheral domains necessarily requires social cooperation. These findings have important implications for targeted interventions, as they suggest that social and contextual changes may more readily shape more public-facing aspects of acculturation whereas interventions at the individual level may be more sufficient for private aspects of acculturation.

In spite of relative differences in strengths and these notable exceptions, pathways to acculturation outcomes remain relatively constant across life domains. Notably, both proposed intermediary factors—PSOC, intergroup anxiety—help explain how socioecology influences acculturation. While both negative and positive interpersonal experiences shape acculturation, their effects can be explained through these intermediary variables. Specifically, although experiences of prejudice were negatively associated with cultural change and positively associated with cultural maintenance, these relationships were fully mediated by PSOC, and in the case of cultural change, intergroup anxiety. Aligned with other studies (e.g., Dandy & Pe-Pua, 2013; Valdez et al., 2013), experiences of prejudice may lead immigrants to not sense membership and instead feel negatively towards the receiving community, prompting them to turn away from it and turn more solely to the ethnic immigrant community to find belonging and fulfill their needs, impeding cultural change while promoting cultural maintenance. This pattern aligns with research on rejection-identification and rejection-disidentification, which suggests that perceived exclusion from a national group can lead to greater affiliation with one’s racial/ethnic or social group and psychological withdrawal from the national community (Branscombe et al., 1999; Jasinskaja-Lahti et al., 2009). In most cases, the same is true for the quality of contact with these groups—though naturally in the opposite direction; positive contact with U.S.-born community members and Latine immigrants is associated with cultural change and maintenance, respectively, vis-à-vis PSOC with each group, and in the case of cultural change, decreases in intergroup anxiety, fitting with the contact hypothesis (Allport, 1954).

As such, PSOC and intergroup anxiety present prime intervention targets. For example, while working to decrease prejudice in the overarching receiving community is critical, since people can experience PSOC in multiple communities, integrating immigrants into positive relational sub-communities within receiving communities can support PSOC development (Brodsky et al., 2002; Buckingham et al., 2018a; Mannarini et al., 2018), aligning with welcoming movement strategies (Welcoming America, 2016). Furthermore, reducing intergroup anxiety and fostering mutual belonging may be achieved through structured intergroup dialogs, community-based projects, or shared civic efforts that build equal-status, cooperative relationships and are supported by local policies and institutional resources (Dessel & Rogge, 2008; Gurin et al., 2013; Pettigrew & Tropp, 2008; Stolle & Harell, 2013). Whereas positive and meaningful contact can improve intergroup relations, negative or superficial contact may reinforce prejudice (Dixon et al., 2005). Thus, communities should complement these approaches with structural strategies that target threat and institutional barriers, as research shows that policy reforms, anti-discrimination initiatives, and equitable access to resources are essential for fostering immigrant integration and inclusion (Bloemraad, 2006; Cheung & Phillimore, 2014; Huang & Liu, 2018; Mbise et al., 2022). Programs that foster cross-cultural learning and sharing may be especially useful (e.g., Goodkind, 2006; Goodkind & Hess, 2017; Goodkind et al., 2024).

Other aspects of the socioecology appear to have a more direct impact on acculturation. For example, while receiving communities’ preferences for cultural change and maintenance were associated with Latine immigrants’ acculturation through PSOC, the mediation was only partial. It is likely that while some of these perceived acculturative preferences were welcomed, others may have been seen as demands for compliance. This is perhaps especially true in peripheral domain cultural change where others’ preferences had no bearing on PSOC. Research has shown that people, particularly those with less sociopolitical power, can feel forced to change and maintain their cultural practices in ways they do not wish (Buckingham & Suarez-Pedraza, 2019; Chirkov, 2009; Fedi et al., 2019; Horenczyk et al., 2015). Consequently, we should explore how these preferences are perceived and received to understand their implications.

Finally, the more Latine immigrants felt their culture was being threatened by the receiving community, the less they made cultural changes due to experiencing more intergroup anxiety and less PSOC with them, and the more they maintained their original cultures as well. Symbolic threat is often studied in members of a culture with more power and privilege but can and does exist across other groups, as demonstrated here. Addressing symbolic threat is an important area of further research to determine appropriate interventions. Some threats will likely necessitate structural changes while others may be addressed at individual levels; for example, psychotherapeutic interventions may decrease the perception of symbolic threats (Birtel & Crisp, 2015).

### 4.1. Limitations and Future Directions

These findings must be considered within the context of the study’s limitations. First, the cross-sectional design lacks temporal precedence and thus directionality is only theorized. Participants were required to recall their past perspectives to report on acculturation preferences (‘ideal’ acculturation), and thus their perceptions of the past are inherently shaped by their current experiences. For example, current levels of PSOC may have influenced how immigrants reconstructed their desired acculturation trajectories. Longitudinal research is needed to determine the directionality of hypothesized relationships.

Data are inherently impacted by study measures and methods. Data were collected during Trump’s first presidential campaign and thus reflect the sociopolitical climate of a period marked by heightened nationalist rhetoric, economic anxieties, and fears about immigration. Participants’ ability to accurately reflect on their own attitudes and experiences may be limited. Responses may have also been shaped by assumptions participants made about the researchers, the research, and what would be socially desirable. Bilingual non-immigrants and 1st and 2nd generation Latine immigrants collected data, surveys could be responded to anonymously through a waiver of written consent, and partnerships with local community organizations supported research in ways that were more likely to be acceptable to participants so as to minimize the possibility of data alteration.

Over 200 definitions of culture were noted in the literature three decades ago (Lonner, 1994). The RAEM (Navas et al., 2005) defines culture very broadly, allowing participants to consider what makes up culture in particular categories. This inclusive approach presents measurement challenges, as responses represent immigrants’ own conceptions of their heritage cultures and their receiving community’s cultures, which are likely viewed as the cultures of those who hold the most power in receiving communities (Chirkov, 2009). Qualitative studies that deconstruct culture to examine specific aspects of cultural life that participants themselves identify as meaningful are vital (e.g., Buckingham & Brodsky, 2015; Berry, 2019; Chirkov, 2009; Matsudaira, 2006) as they provide practical insights for developing targeted interventions across socioecological levels.

While diverse participants were recruited and their demographics aligned well with the population demographics of their locations, due to the stratified snowball sampling method, sample representativeness cannot be guaranteed. Given the diversity within Latine immigrants and across U.S. receiving contexts, caution must be exercised when generalizing findings. Future research should explore whether these findings replicate across other sociopolitical climates and geographic regions, particularly using nested designs that can account for variability across neighborhoods, cities, or states. This would allow for a more nuanced understanding of how local and regional contexts shape acculturation.

Within this sample, findings suggest that, in large part, the impacts of socioecology on acculturation can be explained through PSOC and intergroup anxiety. Thus, future research should pull from social and community psychology to identify methods for increasing PSOC with multiple communities and decreasing intergroup anxiety. Still, at their most predictive, more than half of the variance in acculturation outcomes remains unaccounted for by these proposed models, suggesting that many other factors play a role in acculturation. Qualitative findings from this study have shown that a variety of community-level resources—for example, education, media, social services, support groups, grocery stores, nightlife, transportation, technology—are critical for both cultural maintenance and change. The field would benefit from the examination of targeted resources on acculturation outcomes over time. Finally, while this study focused on intergroup anxiety in relation to the receiving community, future research might explore whether intergroup anxiety is experienced in relation to one’s ethnic immigrant community, particularly in light of recent research on bicultural stress and intragroup marginalization and discrimination.

### 4.2. Conclusions

This study demonstrates that socioecology better accounts for acculturation outcomes than individual desire alone, pointing to numerous community-level intervention points. Acculturation science has done well mapping out the numerous individual-level factors that shape acculturation. While attention has turned to interpersonal and contextual factors in the past decade, much work remains. Future research should continue to examine malleable social and contextual variables that shape acculturation. As we develop acculturation research in the context of increasingly nationalistic, xenophobic, and insular regimes (Walia, 2021), a focus on socioecology is vital. The system-level interventions such research can identify have the potential to shape numerous lives.

## Figures and Tables

**Table 1:** Sample demographics.

		*M*	*SD*
Age		37.91	12.93
Years in the United States		16.56	9.50
Proportion of Life in U.S.		44.8%	22.4%
Region of Origin			
	Mexico	52.0%	
	Central America	25.0%	
	South America	19.8%	
	Caribbean Islands	3.2%	
Gender			
	Woman	60.6%	
	Man	38.2%	
	Other	1.2%	
Level of Education			
	None–8th grade	20.5%	
	9th grade–Diploma/GED	32.5%	
	Some College–Bachelor’s	37.5%	
	Postgraduate Degree	9.5%	
Employment Status			
	Employed	66.5%	
	Unemployed	7.8%	
	Other (Homemaker, Student, Retired, Disabled)	25.7%	
Immigration Status			
	U.S. Citizenship	39.2%	
	Permit	29.9%	
	No Authorization or No Answer	30.9%	

**Table 2:** Means and standard deviations of study variables.

Variable		*M*	*SD*
Ideal Cultural Change		10.33	2.38
	Peripheral	3.55	0.87
	Intermediate	3.56	1.11
	Central	3.23	1.00
Real Cultural Change		9.97	2.51
	Peripheral	3.49	0.89
	Intermediate	3.35	1.11
	Central	3.14	1.00
Ideal Cultural Maintenance		11.14	2.23
	Peripheral	3.19	0.90
	Intermediate	3.82	1.04
	Central	4.13	0.86
Real Cultural Maintenance		9.98	2.35
	Peripheral	2.83	0.91
	Intermediate	3.39	1.07
	Central	3.75	0.93
Perceived Cultural Change Preferences of Receiving Community		10.43	2.79
	Peripheral	3.69	1.00
	Intermediate	3.42	1.16
	Central	3.32	1.04
Perceived Cultural Maintenance Preferences of Receiving Community		8.14	2.73
	Peripheral	2.51	0.98
	Intermediate	2.72	1.09
	Central	2.91	1.05
PSOC with Receiving Community		57.63	15.65
PSOC with Latine Immigrant Community		60.49	14.59
Intergroup Anxiety		52.71	20.54
Prejudice		17.51	12.21
Quality of Contact with U.S.-born People		14.25	3.21
Quality of Contact with Latine Immigrants		15.85	2.95
Symbolic Threat		59.10	14.46

**Table 3:** Overall cultural change path analysis test statistics of direct effects.

	PSOC Receiving Comm.		Intergroup Anxiety		Real Cultural Change	
	*t (p)*	Partial *r*	*t (p)*	Partial *r*	*t (p)*	Partial *r*
Step 1						
Immigration Status	4.75 (*p* < 0.001)	0.23	−3.88 (*p* < 0.001)	−0.19	6.95 (*p* < 0.001)	0.33
Time in the U.S.	1.88 (*p* = 0.062)	0.09	−2.63 (*p* = 0.009)	−0.13	2.15 (*p* = 0.032)	0.11
Ideal Cultural Change	6.11 (*p* < 0.001)	0.29	−4.23 (*p* < 0.001)	−0.21	9.40 (*p* < 0.001)	0.42
Step 2 ^a^						
Perceived Preference for Cultural Change	2.94 (*p* = 0.003)	0.15	−1.55 (*p* = 0.121)	−0.08	6.04 (*p* < 0.001)	0.29
Prejudice	−2.18 (*p* = 0.030)	0.11	5.28 (*p* < 0.001)	0.26	−0.08 (*p* = 0.504)	−0.00
Quality of Contact with Receiving Community	7.52 (*p* < 0.001)	0.35	−7.50 (*p* < 0.001)	−0.35	1.37 (*p* = 0.170)	0.07
Symbolic Threat	−3.84 (*p* < 0.001)	−0.19	2.31 (*p* = 0.021)	0.12	0.16 (*p* = 0.872)	0.01
PSOC with Receiving Community	—	—	—	—	3.68 (*p* < 0.001)	0.18
Intergroup Anxiety	—	—	—	—	−2.87 (*p* = 0.004)	−0.14

^a^ Time in the U.S., immigration status, and ideal cultural change were also included in Step 2.

**Table 4:** Overall cultural change path analysis test statistics of indirect effects ^a^.

	Through PSOC: Receiving Comm.		Through Intergroup Anxiety	
	***t***	***p***	***t***	***p***
Perceived Preferred Cultural Change	2.24	0.013	−1.32	0.093
Prejudice	−1.81	0.035	−2.56	0.005
Quality of Contact with Receiving Community	3.26	<0.001	2.75	0.003
Symbolic Threat	−2.59	0.005	−1.76	0.039

^a^ Controlling for time in the U.S., immigration status, and ideal cultural change.

**Table 5:** Overall cultural maintenance path analysis test statistics of direct effects.

	PSOC Receiving Comm.		Real Cultural Maintenance	
	*t (p)*	Partial *r*	*t (p)*	Partial *r*
Step 1				
Ideal Cultural Maintenance	6.32 (*p* < 0.001)	0.30	12.59 (*p* < 0.001)	0.53
Step 2 ^a^				
Perceived Preference for Cultural Maintenance	5.50 (*p* < 0.001)	0.15	3.40 (*p* < 0.001)	0.17
Prejudice	4.48 (*p* < 0.001)	0.11	−0.21 (*p* = 0.832)	−0.01
Quality of Contact with Latine Immigrants	5.59 (*p* < 0.001)	0.35	−0.40 (*p* = 0.693)	−0.02
Symbolic Threat	−1.45 (*p* = 0.149)	−0.19	2.08 (*p* = 0.039)	0.10
PSOC with Latine Immigrant Community	—	—	7.56 (*p* < 0.001)	0.35

^a^ Ideal cultural maintenance was also included in Step 2.

**Table 6:** Overall cultural maintenance path analysis test statistics of indirect effects ^a^.

	Through PSOC: Immigrant Comm.	
	*t*	*p*
Perceived Preferred Cultural Maintenance	4.43	<0.001
Prejudice	3.81	<0.001
Quality of Contact with Latine Immigrants	4.47	<0.001
Symbolic Threat	−1.41	0.080

^a^ Controlling for ideal cultural maintenance.

## Data Availability

Data are available from the corresponding author upon request.

## Notes

1 Immigration status was treated as a 3-point ordinal variable where 0 = no authorization, 1 = legal status, and 2 = naturalized citizenship.

2 No demographic variables were included as none were significantly correlated with the outcome of interest.

3 Immigration status and time in the U.S. were controlled for in the domain-level cultural change models; Domain models and test statistic tables are not included due to space constraints. They are available upon request.

## Abbreviations

The following abbreviations are used in this manuscript:

PSOC	Psychological sense of community
RAEM	Relative acculturation extended model
US	United States
